# Editorial: Women in lipids in cardiovascular disease

**DOI:** 10.3389/fcvm.2025.1547089

**Published:** 2025-01-23

**Authors:** Irena Levitan, Catherine Martel

**Affiliations:** ^1^ Department of Medicine, Pulmonary Section, University of Illinois Chicago, Chicago, IL, United States; ^2^ Department of Medicine, Faculty of Medicine, Université de Montréal, Montreal, QC, Canada; ^3^ Montreal Heart Institute, Montreal, QC, Canada

**Keywords:** women in science, LDL, HDL, lipoprotein metabolism, lipophagy

**Editorial on the Research Topic**
Women in lipids in cardiovascular disease

## Introduction

The pursuit of science for a long time has been open exclusively to men, with a general belief that women are incapable and uninterested (1). The first woman to receive a medical degree in the United States was Elizabeth Blackwell, who was admitted to Harvard in 1847 and became a trailblazer for women in medicine (2). The legend is that Blackwell was inspired by a dying friend who believed that she might have been better treated by a woman physician. Today, women became a majority in the US medical schools as medical students, but challenges remain. Based on The Association of American Medical Colleges report on *The Gender Gap at Medical Schools in the United States*, while women comprise more than 50% of the medical students, women representation among full professors is still less than 30%. Notably, though the gender gap is progressively decreasing. In this collection, we highlight the contributions of four teams led by exceptional women scientists to the field of lipids and cardiovascular disease.

Daisy Sahoo, Dean of the School of Graduate Studies and Women in Science Endowed Professor focuses her research on the roles of scavenger receptors in lipoprotein metabolism, atherosclerosis, diabetes and obesity. In this issue, Bobek et al. explore how dysfunctional HDL contributes to atherosclerosis. Epidemiological studies show that high HDL-cholesterol levels are linked to a lower risk of cardiovascular disease (CVD). However, not all HDL particles are the same—for instance, those from patients with coronary artery disease have reduced anti-inflammatory and antioxidant functions. Given the lack of clinical benefits from therapies aimed at increasing HDL-cholesterol, assessing HDL function may offer a better measure of CVD risk. Building on their previous research, Sahoo group found that lipoproteins, in the presence of the ER stress inducer thapsigargin, synergistically increase ER stress markers. Both native and modified HDL had similar effects on ER stress as oxLDL.

Mireille Ouimet, Associate Professor at the University of Ottawa, focuses on lipophagy, a novel pathway that degrades fat, and its role in atherosclerosis. In the current issue, Robichaud et al. emphasize the importance of sex-specific approaches in developing cardiovascular therapies. In this study, the team used trehalose, a transcription factor EB (TFEB) activator, to explore the role of autophagy in a mouse model of atherosclerosis regression. They report a sex-specific effect of trehalose, where it reduced lipid content, inflammation, and increased collagen in female mice but not in males. Additionally, the team identified intrinsic differences in autophagy flux between sexes, with female mice exhibiting lower plaque autophagy than males. The investigators suggest that these differences likely make female mice more responsive to atherosclerosis regression.

Wann Jia Loh, a consultant endocrinologist working in Changi General Hospital, Singapore, where she leads the Lipid Service. In this issue, Jia Loh et al. evaluated whether measuring the remnant cholesterol carried by triglyceride-rich lipoprotein fraction provides an efficient tool to access cardiovascular risk factor in patients with well controlled LDL levels. The motivation of the study is to develop criteria to assess the residual cardiovascular risk in patients who achieved the recommended levels of LDL cholesterol. In a 2-year retrospective study that included more than 20,000 patients, the study finds that this is not an efficient strategy for evaluating residual cardiovascular risk except in patients with severe hypertriglyceridaemia. Further studies are needed to establish the appropriate guidelines to diminish the residual cardiovascular risk.

Jingen Li, a cardiologist at Beijing University of Chinese Medicine and her team explore the role of Chinese herbal medicine in lowering blood cholesterol levels and improving cardiovascular outcomes. Analyzing 23 trials which included more than 7,000 participants, Xie et al. discover that supplementing standard western medicine with Chinese herbal therapy resulted in a significant further decrease of plasma cholesterol and triglyceride levels, which was associated with a decreased cardiovascular risk.

Taken together, these studies provide significant novel insights into lipoprotein research, both in terms of the basic science and clinical translational studies.

## Authors highlight

Dr. Wann Jia Loh graduated from Imperial College London with Distinction and is a recipient of several awards, including Population Health New Investigator award and Nurturing Clinician Researcher Award. Her research and clinical interests include dyslipidaemia, obesity, atypical diabetes, severe insulin resistant states, and women's health. Dr. Loh is a mother of 2 young children with a busy schedule and stress that includes clinical work, scientific pursuits and ensuring her family has good quality time and compensate for lack of time. She expresses gratitude for the support and understanding of her family, friends, kind neighbors, team members, and colleagues who helped her in various ways over the years. In her own words, “*It takes a village to support a woman clinician scientist*”. Dr. Loh's colleague, Dr. Mon Tun, is a Senior Analyst at Changi General Hospital, Singapore, trained in epidemiology and health technology assessment. With a background in population health, infectious disease epidemiology, and healthcare innovation, she focuses on improving healthcare delivery through research on disease burden, cost-effectiveness, and predictive analytics. In her own words: “*While balancing a busy career, I am deeply inspired by the support from my colleagues and mentors, which fuels my passion for improving healthcare systems and outcomes*.”

## Notes from the editors

**Irena Levitan** is a professor at the University of Illinois, Chicago focusing her research on vascular biophysics, which includes studies of endothelial biomechanics and membrane properties. As an American-Israeli scientist, she believes in the importance of dialogues between people with different languages, cultures and identities. She also believes that women who are not only excellent scientists but also excel in emotional intelligence should play a major role in creating and supporting an inclusive and diverse scientific endeavors. This issue is our current contribution. **Catherine Martel** is an associate professor at the Université de Montréal, a French-speaking institution in Quebec, Canada. While Quebec takes pride in preserving the French language, women in science, like many researchers, often face additional challenges when navigating the predominance of English in scientific communication. For non-English speakers, translating complex ideas into research proposals or articles can be daunting, particularly when cultural nuances and writing styles may differ from dominant norms. Women, who are already underrepresented in some fields, may feel an added burden to overcome these linguistic and cultural barriers. Although certain funding agencies allow proposals to be submitted in French, this can restrict access to feedback from English-speaking reviewers, potentially limiting opportunities for collaboration and success. Similarly, publishing in scientific journals often requires a strong command of English to ensure ideas are clearly conveyed and widely recognized. Addressing these issues is essential to supporting women in science and fostering a more inclusive, equitable environment that values diverse languages and perspectives.

## Concluding remarks

We are delighted and proud to have presented The Frontiers in Cardiovascular Medicine “*Women in lipids in cardiovascular disease*” series of article collections, which highlight the work of female scientists in the full range of lipid research and its connection to cardiometabolic disorders. Of particular interest, the first and/or last author of every publication is a researcher who identifies as woman. We hope that this succinct but important scientific contribution will positively influence the promotion of gender and sex parity in research and encourage women and girls to thrive in STEM professions.
